# Arteriovenous malformation presenting as a lip swelling

**DOI:** 10.1002/ccr3.7736

**Published:** 2023-11-19

**Authors:** Ashok Kumar Ariboyina, Ozgur Tatli, Lakshmi Goriparthi, Narendra Achanta, Suresh Kumar Thirumoothy, Suha Turkmen

**Affiliations:** ^1^ Department of Emergency Medicine Hamad Medical Corporation Doha Qatar; ^2^ Al Mashaf Health Center Primary Heath Care Corporation Doha Qatar; ^3^ Department of Surgery Hamad Medical Corporation Doha Qatar; ^4^ Department of Emergency Medicine Qatar University Doha Qatar

**Keywords:** arteriovenous malformations, emergency medicine, lip

## Abstract

**Key Clinical Message:**

Arteriovenous malformations (AVM) are extremely rare on the face and especially on the lip. Lip can be more easily exposed to traumas due to their anatomical location. Especially superficial AV malformations are very susceptible to trauma and can bleed very seriously after being exposed to such effects. AVMs management generally based on their hemodynamic characteristics and growth modalities. The surgical treatment requires elaborate planning and multidisciplinary approach. When evaluating a mass with a clinical manifestation of lip swelling, ruling out a vascular anomaly before any intervention is also crucial.

**Abstract:**

Arteriovenous malformations (AVM) are abnormal fistulas between an artery and a vein, without an intervening capillary bed. AVM may occur everywhere in the body, with the brain being of particular concern, as AVM can be complicated by bleeding. Herein, we present the case of a patient with a very atypical AVM location consisting of a lip mass. Given the possibility of hemorrhage and airway obstruction, early detection and timely intervention are mandatory.

## 1 CLINICAL IMAGE

**FIGURE 1:**
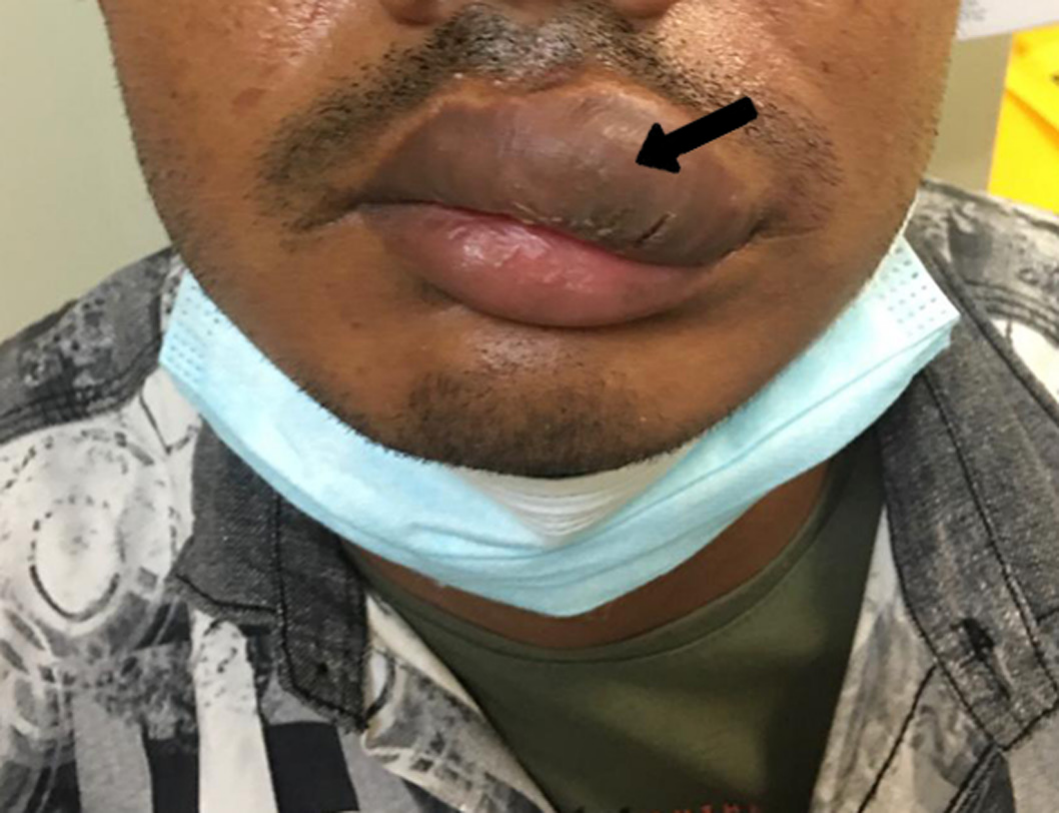
Photograph of the patient showing swelling of the upper lip (black arrow).

**FIGURE 2:**
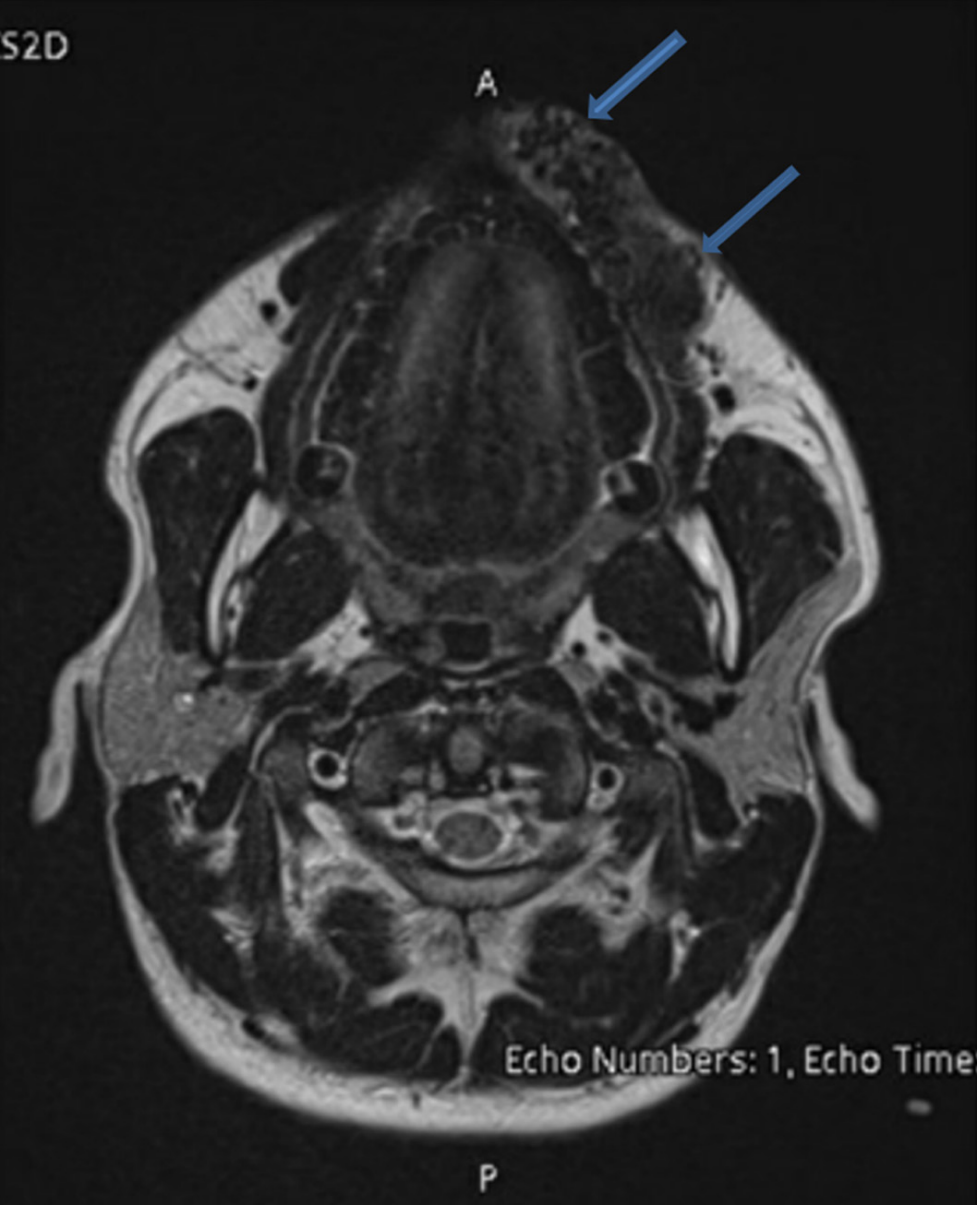
Magnetic resonance imaging (MR) of the head and neck (axial plane, T2‐weighted image) showing a diffuse linear‐tubular signal void areas located in the soft tissue of the lip at the level of the alveolar process (blue arrows).

**FIGURE 3:**
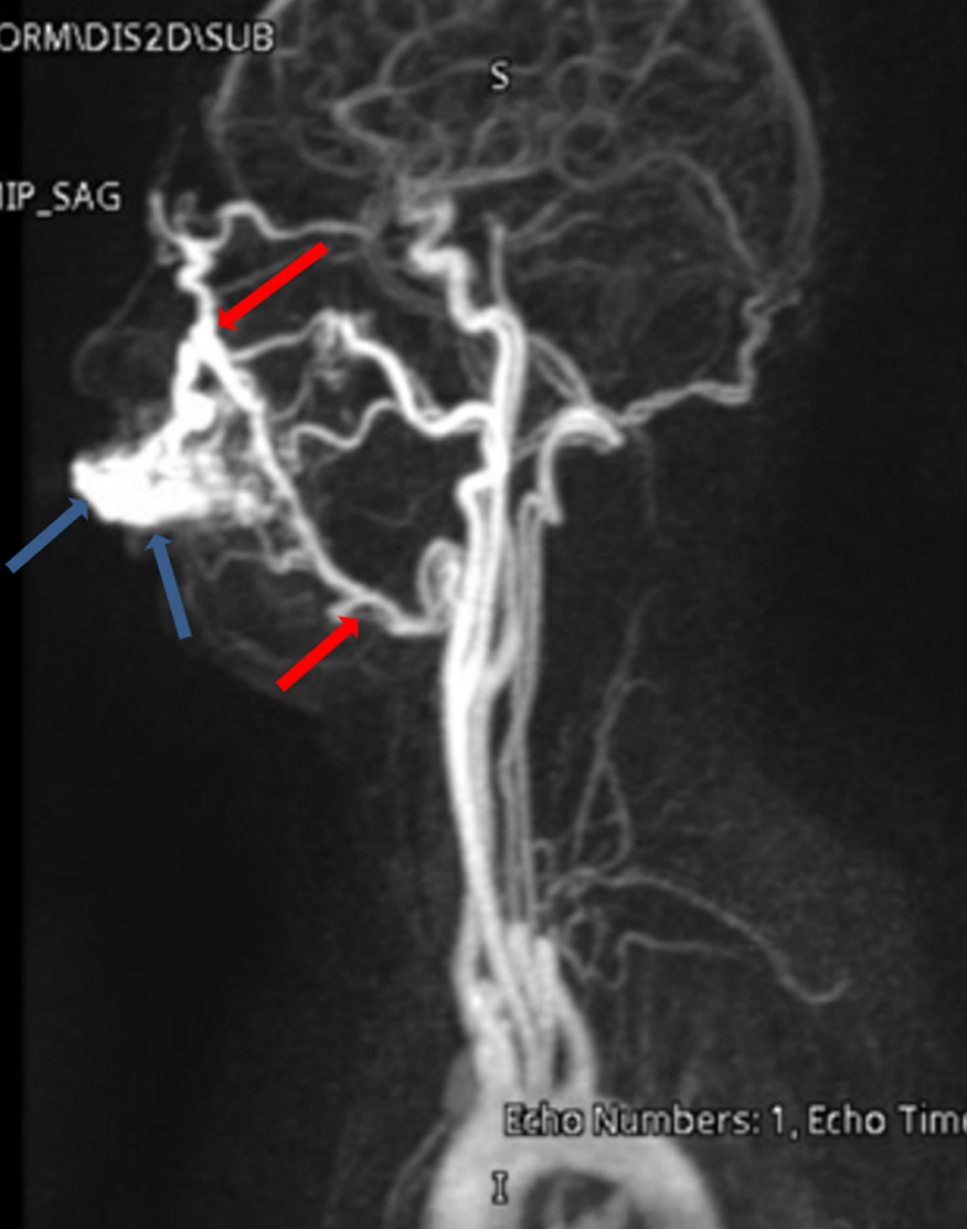
Head and neck MR angiography (coronal plane, maximum intensity projection) showing a vascularized mass (arteriovenous malformation, blue arrows) in the upper lip with arterial supply coming from the distal branches of the external carotid artery (MIP images‐blue arrows).

**FIGURE 4:**
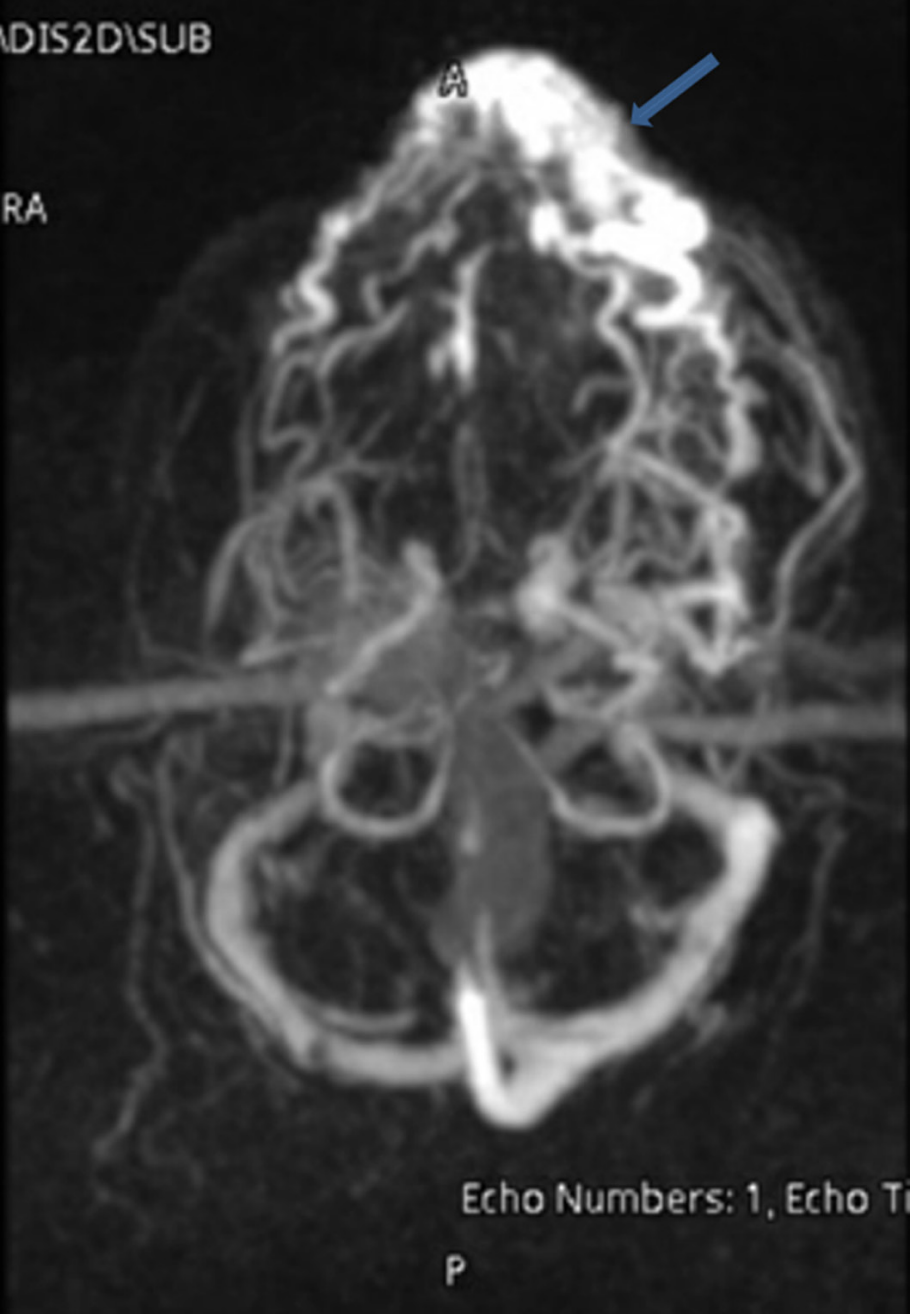
Head and neck MR venography (axial plane, maximum intensity projection) showing a vascularized mass in the upper lip draining into the venous system (MIP images‐blue arrows).

A 27‐year‐old healthy male patient presented to emergency department with complaint of pulsatile painless mass in his left upper lip. On examination a soft, pulsatile, immobile mass was observed on the upper lip (Figure 1). The mass measured 6 cm × 2.5 cm and had well‐defined borders. It was also non reducible and noncompressible. There was no signs of inflammation such as erythema or discharge surrounding the swelling. It was not detected any significant heat increase, ulceration, bleeding or discoloration during the examination. He stated that the swelling had been growing rapidly especially in the last month. He denied any trauma. Abscesses and vascular tumors were considered in the differential diagnosis. Magnetic resonance imaging (MRI) of the neck (Figure 2), MR neck angiography (Figure 3), and MR venography (Figure 4) revealed a vascularized soft tissue mass of the upper lip. Based on the patient's history and clinical findings, the diagnosis of AVM of the upper lip was made.

AV malformations are extremely rare on the face, particularly the lips. The lips can be more easily exposed to traumas because of their anatomical location and can bleed seriously, posing a risk to the patient because of airway obstruction. Although rare, it is important to include AVM in the differential diagnosis of lip masses.

## 2 DIAGNOSIS

Arteriovenous malformations can be congenital or acquired. Head and neck AVM are rare and occur in up to 0.1% of the population, typically in the brain. Extracranial involvement is present in only 8.1% of head and neck AVM.^1^ Facial AVMs are a subgroup of AVMs with fistulous connections between the draining veins and feeding arteries.^2^ AVMs are classified according to the radiological, histological and clinical appearance of the abnormal vessels. While capillary, lymphatic and venous AVM often shows slow‐flow characteristics, arterial malformations show fast‐flow characteristics.^3^, ^4^ Occasionally, both vascular and lymphatic vessels come together. In such cases, the definition is made combination of these structures, and identification is made by using combined names such as arteriovenous malformation (AVM), lymphaticovenous malformation (LVM) or capillary LVM (CLVM).^5^ The history of patient's lesion and its clinical presentation are very important for diagnosis. AVMs have different histopathological features and clinical symptoms and findings. Many clinicians from different specialties are often involved in the diagnosis and management of these patients. The hemodynamic properties and growth patterns of these masses are very important in determining the treatment modality of AVM.^5^, ^6^ Multimodal treatment methods such as preoperative sclerosing agents or embolization are the traditional approaches used in the treatment of these lesions.^2^, ^5^

## 3 CONCLUSION

Acquired AVMs are quite rare in the lip region. They may result life threatening bleedings, lip ischemia, airway obstruction, and high‐output cardiac failure. Hence, early diagnosis and prompt treatment of such cases is necessary. The surgical treatment requires elaborate planning and multidisciplinary approach. This case report provides an overview of acquired AVMs in the lip.

## AUTHOR CONTRIBUTIONS

**Ashok Kumar Ariboyina:** Investigation; methodology; resources; supervision; validation; visualization; writing – original draft; writing – review and editing. **Ozgur Tatli:** Resources; software; visualization; writing – original draft; writing – review and editing. **Lakshmi Goriparthi:** Resources; supervision; visualization; writing – review and editing. **Narendra Achanta:** Formal analysis; methodology; resources; visualization; writing – review and editing. **Suresh Kumar Thirumoothy:** Methodology; resources; writing – original draft; writing – review and editing. **Suha Turkmen:** Methodology; resources; validation; visualization; writing – review and editing.

## CONFLICT OF INTEREST STATEMENT

The authors have no conflicts of interest or financial disclosures to declare.

## ETHICS STATEMENT

Informed consent was obtained from the patient to publish this case report. All procedures performed in this study involving human participants were in accordance with the ethical standards of the institutional research committee and with the 1964 Helsinki declaration and its later amendments or comparable ethical standards.

## CONSENT

Written informed consent was obtained from the patient to publish this report in accordance with the journal's patient consent policy.

## Data Availability

The data that support the findings of this study are openly available at https://doi.org/10.1002/ccr3.7736.

## References

[1] Hormozi AK , Shafii MR . Supraclavicular flap: reconstructive strategy for massive facial arteriovenous malformations. J Craniofac Surg. 2011;22(3):931‐936. 21558921 10.1097/SCS.0b013e31820fe191

[2] Ramachandra S , Ippagunta LP . Acquired A‐V malformations – a case report. Transworld Med J. 2014;2:183‐186.

[3] Waner M , Suen JY . Hemangiomas and Vascular Malformations of the Head and Neck. John Wiley and Sons; 1999:20‐26.

[4] Petel R , Ashkenazi M . Pediatric intraoral high‐flow arteriovenous malformation: a diagnostic challenge. Pediatr Dent. 2014;36:425‐428. 25303512

[5] Manjunath SM , Shetty S , Moon NJ , et al. Arterivenous malformation of the oral cavity. Case Rep Dent. 2014;2014:353580. 24660070 10.1155/2014/353580 PMC3934311

[6] Maheshwari UM , Sahu SS , Khatu SP . Arteriovenous malformation of the cheek – a case report and review of literature. Bombay Hosp J. 2010;52:241‐243.

